# The National NeuroAIDS Tissue Consortium (NNTC) Database: an integrated database for HIV-related studies

**DOI:** 10.1093/database/bav074

**Published:** 2015-07-30

**Authors:** Matyas F. Cserhati, Sanjit Pandey, James J. Beaudoin, Lorena Baccaglini, Chittibabu Guda, Howard S. Fox

**Affiliations:** ^1^Department of Genetics, Cell Biology and Anatomy,; ^2^Bioinformatics and Systems Biology Core,; ^3^Department of Pharmacology and Experimental Neuroscience, College of Medicine,; ^4^Department of Epidemiology, College of Public Health and; ^5^Fred and Pamela Buffet Cancer Center, Eppley Institute for Cancer Research, University of Nebraska Medical Center, Omaha, NE 68198, USA

## Abstract

We herein present the National NeuroAIDS Tissue Consortium-Data Coordinating Center (NNTC-DCC) database, which is the only available database for neuroAIDS studies that contains data in an integrated, standardized form. This database has been created in conjunction with the NNTC, which provides human tissue and biofluid samples to individual researchers to conduct studies focused on neuroAIDS. The database contains experimental datasets from 1206 subjects for the following categories (which are further broken down into subcategories): gene expression, genotype, proteins, endo-exo-chemicals, morphometrics and other (miscellaneous) data. The database also contains a wide variety of downloadable data and metadata for 95 HIV-related studies covering 170 assays from 61 principal investigators. The data represent 76 tissue types, 25 measurement types, and 38 technology types, and reaches a total of 33 017 407 data points. We used the ISA platform to create the database and develop a searchable web interface for querying the data. A gene search tool is also available, which searches for NCBI GEO datasets associated with selected genes. The database is manually curated with many user-friendly features, and is cross-linked to the NCBI, HUGO and PubMed databases. A free registration is required for qualified users to access the database.

Database URL: http://nntc-dcc.unmc.edu

## Introduction

Despite significant advances in therapies, infections with the human immunodeficiency virus (HIV) still constitute a major source of morbidity and mortality in the twenty first century. The death toll from AIDS has reached 36 million with millions more affected by HIV infection (1). Neurological disorders (neuroAIDS), involving both the central and peripheral nervous systems (CNS and PNS), affect a high proportion of HIV infected individuals. HIV-associated dementia was recognized early in the epidemic and affected approximately one out of six patients with AIDS (2), however with current combination antiretroviral therapy the prevalence of dementia is less than 1 in 40 (3). Despite these advances, HIV-associated neurocognitive disorders (HAND) continue to afflict those with HIV, and an increase in frequency of mild neurocognitive impairment has been noted (3, 4). The neuropsychological pattern of deficits may also be changing with more frequent abnormalities in learning and executive functioning (3). While HIV encephalitis was commonly found pathologically in those with HIV dementia (5, 6), the neuropathological basis of HAND lacks such a link (7). Peripheral neuropathy is also found frequently, although with the advent of cART and the use of drugs with lower neurotoxicity the prevalence of symptomatic neuropathy has declined (8). Due to the impact of these conditions, their changing clinical picture, the now chronic nature of HIV infection, and the special assessments required to study these diseases and the tissues affected by the subsequent pathologies, specialized tissue repositories and databases are warranted to facilitate studies on neuroAIDS.

Several public databases have been developed to house specific data on HIV research, and these include a number of important resources. The Los Alamos National Laboratory hosts three databases (http://www.hiv.lanl.gov/) on HIV genetic sequences (9), molecular immunology and vaccine trials. This resource also provides access to a large number of tools that can be used to analyze these data. The HIV Mutation Browser (http://hivmut.org/) is a database that uses text-mining techniques to extract data on polymorphisms and mutations in the HIV proteome from the available HIV literature (10), whereas the Stanford HIV Drug Resistance Database (http://hivdb.stanford.edu/) is a curated public database for representing, storing and analyzing data associated with HIV drug resistance, important for surveillance and management of infection with drug-resistant viruses (11). The NCBI HIV Human Interaction Database (http://www.ncbi.nlm.nih.gov/genome/viruses/retroviruses/hiv-1/interactions/) contains data on protein-protein interactions between the proteins of human genes as well as human gene knockdowns which affect HIV replication and infectivity (12). However none of these databases provide a focus on the brain or its functions, which requires specialized assessments during life as well as examination after death. These is one existing database, the HIVBrainSeqDB (http://hivbrainseqdb.dfci.harvard.edu/HIVSeqDB/), which is a public resource that contains annotated HIV envelope sequences from brain and other tissues annotated with clinical data (13). However this database is not currently maintained or updated.

The National NeuroAIDS Tissue Consortium (NNTC) was established in 1998 to facilitate access to antemortem and postmortem tissues and fluids (blood, cerebrospinal fluid) for the international neuroAIDS research community (14). The consortium's goals included establishment of a network of brain banks, collection of nervous system tissues in a standardized fashion, and maximization of the information gleaned from the scientific studies of these tissues (15). In addition, a goal of the NNTC was to link the experimental and clinical data pertaining to each sample. Four study sites with tissue banks were established at San Diego, CA; Los Angeles, CA; Galveston, TX; and New York, NY. All four sites are currently actively recruiting and following-up participants. At each site, HIV-infected individuals are contacted for enlisting in the cohort for this resource. Enrollment entails consent for neuromedical and neuropsychological examinations as well as for obtaining the brain soon after death for examination and tissue banking; a focus on individuals with advanced medical disorders facilitates obtaining autopsy specimens. The NNTC collects information on HIV disease severity, CNS and PNS signs and symptoms, comorbid conditions, laboratory values for a range of medical, immunological and virological parameters, and tissue pathological diagnoses.

As of February, 2015, the NNTC tissue bank has specimens from 1119 subjects (881 with HIV, 238 controls). The current cohort being followed consists of 594 individuals (558 with HIV, 36 controls). Since the founding of the NNTC significant advances in treatment have occurred, and the NNTC has been instrumental in documenting the changes in HIV neuropathology in the evolution of HIV infection from an untreatable condition to a treatable chronic disease (7). While recruitment into the cohort is biased towards those with advanced HIV infection, the cohort and specimens also represent part of the current spectrum of HIV infection in the US, with the use of combination antiretroviral therapy resulting in effective viral suppression. Thus, this enables the use of the resource for current important topics such as studies of eradication of HIV from persistent reservoirs, aging with HIV, and pathogenic mechanisms of HAND in the setting of long-term treatment. With the chronic nature of HIV infection and longitudinal study design at four NNTC sites, a rich collection of antemortem and postmortem data have accrued.

To make the collected tissues and their associated experimental and clinical data maximally accessible to researchers, a searchable database that keeps track of the scientific data collected at the four sites as well as the results generated from the use of tissues requested by researchers from NNTC was needed. To fulfill this goal, an accessible database was developed by the NNTC Data Coordination Center (DCC). In this article, we discuss the need and importance of the NNTC-DCC resource, and provide details on the structure and features of our web-based searchable database.

## Aims of the database

**Figure 1.**
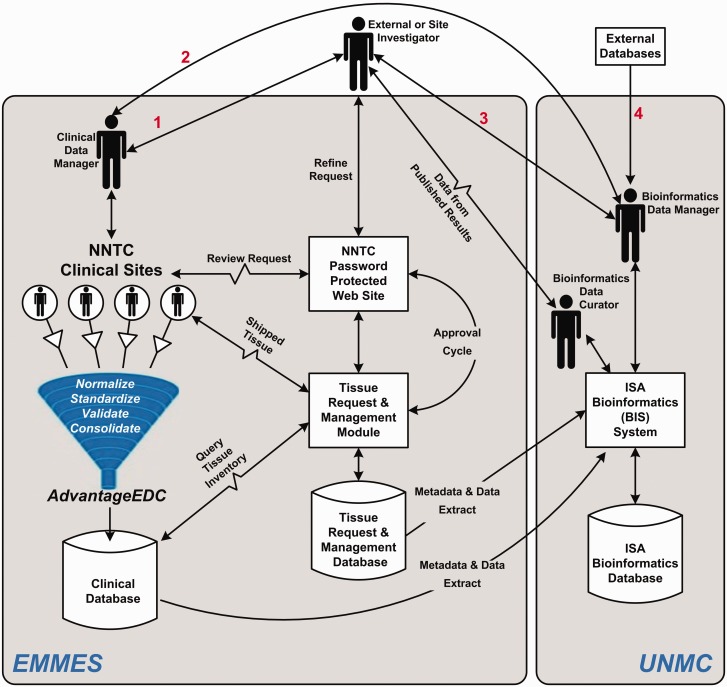
Information and data management flow for the DCC. Investigator requests go through (1) the clinical data manager who works with the investigator and data available through the web site to refine the request, which is then transmitted to the clinical sites and/or (2) the bioinformatics data curator, who can also work with (3) the investigator to refine the request as well as coordinate bioinformatics and epidemiological assistance as needed. Requests are entered into the management system for approval processes, and shipment of specimens/access to data is tracked for completeness. Once studies are complete the data curator works with the bioinformatics data manager to capture the data into the bioinformatics database, which is linked to the clinical database as well as outside databases (4) containing information on NNTC (and potentially other related) experiments.

The NNTC Data Coordinating Center (DCC) was created in 1999 as a liaison between individual researchers and the four tissue banks. To further this goal the National Institute of Mental Health (NIMH) also funded a performance-based contract with the EMMES Corporation (EMMES), which is part of the DCC, to provide data management services and other support activities. In 2013, the University of Nebraska Medical Center (UNMC) joined the DCC. The critical core DCC activities originally included data and tissue request processing and data management support to facilitate the flow of information from NNTC to external investigators (Figure 1). EMMES launched a centralized database containing clinical data and specimen information, established a public web presence, instituted quality assurance processes, and initiated an internal communications platform. The clinical data manager at EMMES has been coordinating tissue requests and the four tissue banks. This includes processing tissue requests from tissue users, which is done via the NNTC password protected website, and entails assignment of tissue request IDs. Beginning in 2013, DCC began to expand its activities to include retrieval of new experimental data generated from tissue requesters and integration of those results and other external information with NNTC. The UNMC bioinformatics data manager and curator are now responsible for obtaining experimental datasets from each study that has used NNTC tissue samples, and transforming these into an accessible and useful format.

As of our knowledge, to date, no dedicated database for neuroAIDS studies exists that contains data in an integrated and standardized form. Therefore, UNMC created a manually-curated, integrated and easily accessible database (hereafter referred to as the NNTC-DCC database) containing experimental data and their metadata from NNTC tissue requesters as well as from other sources, such as the HIVBrainSeqDB. One of the main goals of this database is to provide data (generated by NNTC tissue users) in a centrally standardized data repository format, using the ISA platform (16). The database will serve as a community resource for researchers to keep track of what kind of HIV-related studies have been performed as of date. The database also has a gene search function for genes described in the studies housed in the database with a Gene Search Tool, which is cross-linked with the NCBI’s Gene Expression Omnibus (GEO) database.

### Building of database and web application

Data for each individual study were collected from NNTC tissue requesters and summarized in one or more Excel files. The Excel files contain published and unpublished raw data, as well as a data dictionary where acronyms and terms are defined, as well as a set of published methods detailing how the study was performed.

#### The ISA platform and the ISA-TAB format

ISA is an open-source infrastructure for annotating, managing and sharing data, which is well regarded in the life sciences domain. Some of the salient built-in features of the ISA software that make it an ideal platform for the current project include: (1) platform-independent components that can work on PC, Mac and Linux systems, (2) an extensible, cross-domain format known as ISA-Tab that allows easy storage and conversion to other commonly used formats, (3) a relational database that allows for local management and storage of experimental metadata and interfacing with external public repositories via a web-based query interface and (4) a validation tool to impose data- compliance by checking adherence of data fields and types to pre-defined templates. According to the ISA format, an investigation (I) is a high level concept, grouping together associated studies. Each study (S) contains information on the subjects under it (e.g. characteristics, treatments, tissues). Each study consists of associated assays (A), which includes test(s) performed on the study material and associated data from measurements.

Each study in our database follows the ISA-TAB format (17–19), which was launched by the BioSharing Initiative in 2012, with the goal of streamlining data sharing between thousands of databases, several hundred terminologies, and 120 exchange formats. Studies in ISA-TAB format conform to the principles set forth by the Minimum Information for Biological and Biomedical Investigation (MIBBI) project (20). Studies are archived, uploaded and displayed online using the ISA software suite of the ISA community (16). Then, using ISAcreator configurator, a configuration file was designed for the individual methods and technologies used by the study. Next, the data and their metadata were put into individual archives using the ISAcreator, and loaded into the database using the bii manager.

#### Database framework

The database itself was developed using the BioInvestigation Index web application using Java Server Faces version 2 (JSF2). JSF2 is a widely-used Java-based complete database programming language framework with lots of support. It also comes with many ready to use components, besides allowing new, reusable components to be made. The application server used was Jboss 5.1.0.GA. MySQL version 5.1.73 was used on GNU/Linux to store the data. Ajax, Javascript and jQuery scripts were also employed to facilitate the dynamic content of the website. The basic database framework provided by ISA was extensively modified for NNTC purposes.

#### Entity–Relationship diagram

**Figure 2.**
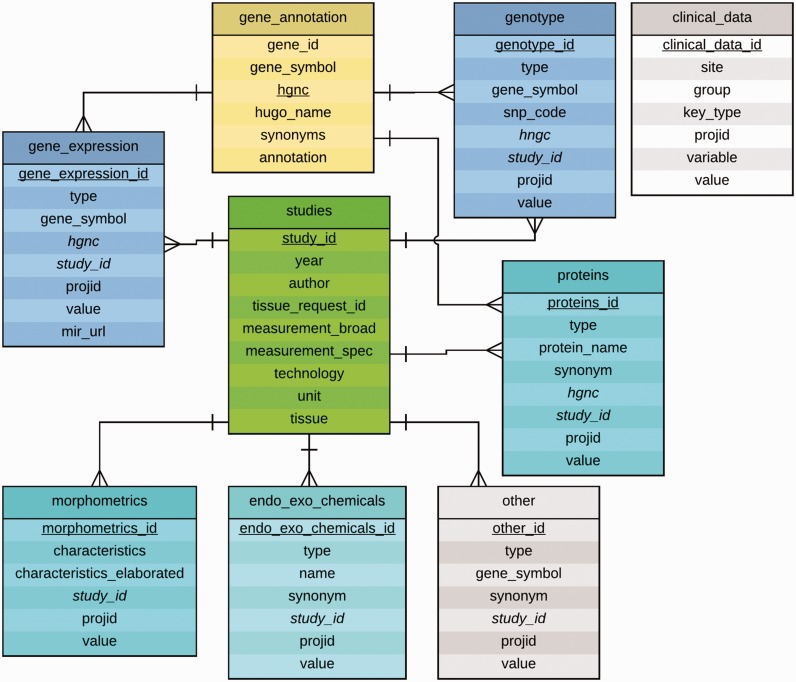
Entity-Relationship Diagram for the experimental data tables in the NNTC-DCC database. The central table is the studies table which describes a given study characterized by a study_id (e.g. in the form of S0001.1), principal investigator, year, tissue request ID, measurement, technology, unit and tissue. A study may consist of multiple tissue requests, thus the study_id might be incremented (e.g. S0001.1 to S0001.2). The tables gene_expression, genotype, proteins, endo_exo_chemicals, morphometrics, and other are grouped around the studies table in a 1:N relationship signifying that a single study may study multiple genes, proteins, morphological characteristics, etc. The table gene_annotation contains HGNC ids, gene symbols, synonyms and annotations for genes and proteins, hence their connection to the tables gene_expression, genotype and proteins. The ER diagram was created by using Lucidchart at www.lucidchart.com.

The entity-relationship (ER) diagram of the current database is shown in Figure 2. Eight MySQL tables were set up that include a ‘studies’ table, a ‘gene_annotation’ table, and six tables for data categories, each representing a different type of experimental data. Each of the data category table uses a secondary key called ‘study_id’ to connect to the ‘studies’ table that contains a description of each of the studies stored in the database. Each table also contains measurement values for each patient, represented by a column called ‘projid’. The table gene_annotation contains a field called gene_symbol by which it can be referenced by entries in the gene_expression, genotype, and proteins tables. The column hgnc references the corresponding gene in the HUGO database, and when information for each gene is displayed, a link is provided to the gene’s page.

### Database content

The data in the database originate from tissue requesters’ research experiments. Experimental data for 95 studies covering 170 assays were collected from 61 individual principal investigators (see Supplementary Excel file). The whole process starts with a tissue request made by the tissue requester to the NNTC via the NNTC website. Brain tissues and/or biofluids are then relayed from the four tissue banks (clinical sites), and delivered to the tissue requesters who use them in their research studies. According to the agreement between NNTC and the requesters, experimental data generated from these studies should be made available back to the NNTC and transmitted to the bioinformatics data manager and curator. Each tissue request is tracked by a study_id and the corresponding experimental data are formatted into an ISA-TAB study and archived using the ISAtool into the NNTC-DCC database. Each study may consist of both published and unpublished data. Data for each study are available by clicking on the link to the study in the browser section on the website, and then clicking on the ‘Raw Data’ link to access and download the data.

### Database statistics

**Figure 3.**
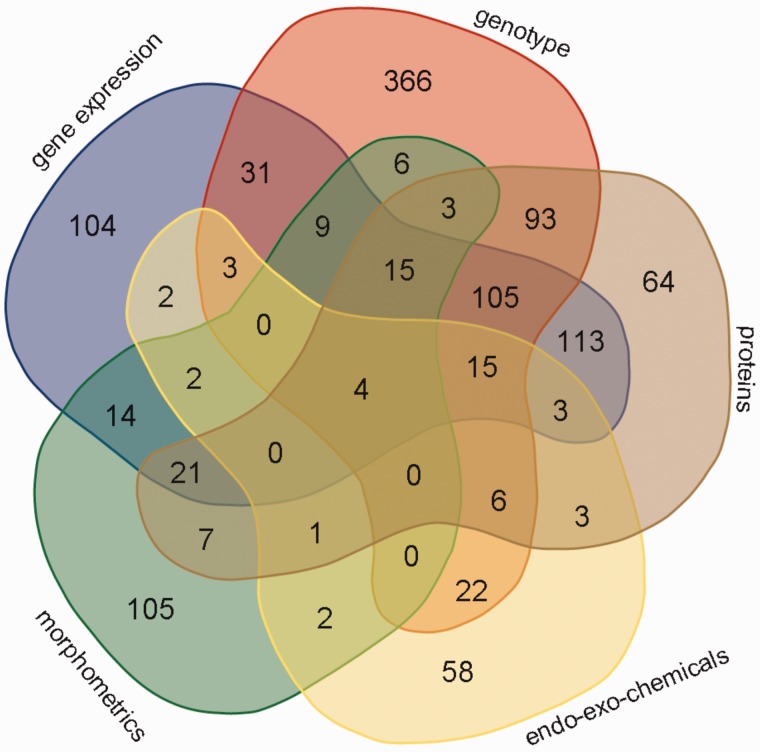
Venn diagram showing the number of patients with data points from different combinations of experimental data categories. These categories include gene_expression, genotype, proteins, endo_eco_chemicals (drugs, endotoxins, glycosaminoglycans and steroids) and morphometrics. The data category other was excluded, because the Venn diagram can depict five categories at most; the category other had few data points. The Venn diagram tool is available at http://bioinformatics.psb.ugent.be/webtools/Venn/.

Experimental data comprising six main categories (gene expression, genotypes, protein levels and enzyme activity, endo-exo-chemical levels, morphometrics and other) were collected from 1206 patients. The categories, subcategories, number of data points, number of genes, proteins, patients with data per subcategory and the number of studies are shown in Table 1. The data in this table represent a snapshot of the database as of April 20, 2015. Figure 3 is a Venn diagram showing the number of patients with different data categories and in different combinations of these categories. Notably there is substantial overlap between patients with gene expression and genotype data (61 patients) as well as gene expression and protein data (107 patients). Overall there are 109 patients with data points from all three of these experimental data categories.

**Table 1.** Classification of experimental data types in the NNTC-DCC database

Data Category	Data Subcategory	Number of data points	Number of entities studied	Number of patients	Number of studies
Gene expression	RNA (non-microarray)	4687	35	448	11
	RNA (microarray)	15 367 490	343 745 probe IDs	86	5
Genotype	SNP	17 560 135	21 806	705	5
	VNTR	1026	3	342	1
	PNGS	13	1	5	1
	Average protein length	39	3	5	1
	Average positive charge	39	3	5	1
	Mutation	13	1	5	1
Proteins	Enzyme activity	486	6	66	3
	Protein levels	3625	95	461	20
Endo-exo-chemicals	Drugs	134	2	28	2
	Endotoxins	212	1	81	4
	Glycosaminoglycans	12	1	12	1
	Steroids	30	1	15	1
Morphometrics	Arterial characteristics	79 044	43	189	1
Other	DNA mutation levels	24	1	12	1
	DNA levels	240	3	204	3
	FACS data	5	1	5	1
	Macrophage infectivity	34	1	6	1
	Albumin/creatinine ratio	30	1	30	1
	Pathological analysis	89	1	85	1

Entities cover gene names, protein names, SNP/VNTRs, chemicals, arterial characteristics and other items named in the data subcategory. SNP = Single Nucleotide Polymorphism. VNTR = Variable Number of Tandem Repeats. PNGS = Potential N-linked Glycosylation Site.

A large amount of data originated from several of the studies performed on NNTC samples. Over 17 million data points come from a genomic SNP experiment (21). This study produced massive data on polymorphisms that influence gene expression in general. Another large number of data points, over 15 million, come from 5 gene expression experiments using microarrays and real time PCR (21–25). These studies include analysis of gene expression changes in mRNAs and miRNAs associated with neurocognitive impairment as well as networks of miRNAs after neuronal exposure to HIV. Yet another experiment generated over 79 thousand data points comprise morphometric data from an analysis on the size, position, and morphological characteristics of arteries and their importance in neuroAIDS (26).

All experimental data entered has been examined to verify de-identification for patient confidentiality and HIPAA-compliancy.

### Data access and user interface

#### Registration

The database can be accessed at http://nntc-dcc.unmc.edu. New users may register on the front page after clicking the Login button. The user must provide their first and last names, title, affiliation, email address, username, password, and describe why they would like to access data from NNTC. Subsequently, the user provided information will be verified by the NNTC-DCC personnel and registrations are approved manually. After acceptance, a confirmation email is sent to the new user. Users who have forgotten their password may reset it by clicking the ‘forgot password’ link, which will redirect them to another screen, where a new password may be set after supplying their email address, last name and username as a verification of the user’s identity.

**Figure 4.**
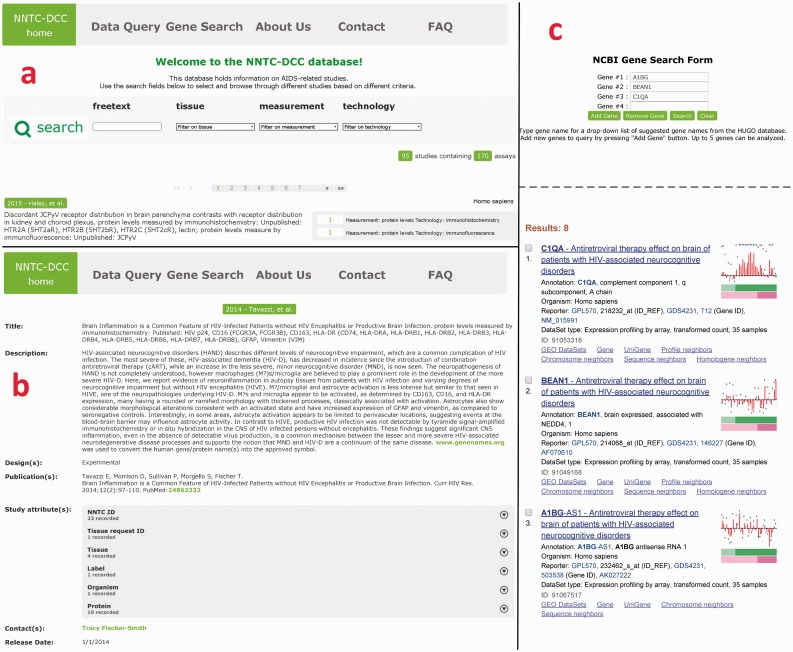
Navigation of the NNTC-DCC database. After signing up and logging in, the user arrives at the browser (a), where studies can be selected based on a freetext search by entering keywords, or by filtering based on tissues, measurement and/or technology. Studies which meet the search/filter criteria show up below the search bar, ordered by date. Each study is characterized by a title, short description and measurement/technology. By clicking on the title the user comes to the study page (b), where full details of the study are displayed, e.g. long description, design, contacts, etc. By clicking on the ‘Gene Search’ link on the top tab, the user will then come to the gene search page (c). The user can search for up to 5 genes from a drop-down box populated by a list of genes names from the HUGO database. When the Search button is clicked, an NCBI page pops up with GEO sets associated with these genes.

Upon logging in, the user is directed to the main page of the database, which displays descriptions of the studies, a browser panel and a navigation panel (Figure 4a). Each investigation covers several studies, which in turn employ several assays and technologies. Each study is listed in the browser with a title (author and year), a short description, and the number and types of assays employed. The studies are ordered chronologically in the browser, 15 per page.

#### Browsing the database

The user can browse among studies using the browser panel by entering a free-text term, or by choosing from a combination of 76 tissue types, 25 measurement types and 38 different technologies. The tissue types, measurement types, and technologies are listed in the Supplementary File 1. In the free-text search, logical operators such as ‘&’, ‘|’, ‘not’ can be used for more complex queries. The free-text search scans through the entire text of all of the fields in all of the studies.

The studies in the NNTC-DCC database follow the hierarchical convention laid down in the ISA format: By clicking on the title of a study listed in the browser, the user can access a full description for each study on a new page, which displays the information listed below. Figure 4b shows the detailed study page for a study done by Tavazzi et al. (27).

- **Title:** Title of paper
- **Description:** Full description of study, including study goals and what was studied and how the study was performed
- **Design(s):** Design of the study
- **Publication(s):** Full reference to paper that the study was described in, including PubMed ID and link
- **Study attribute(s):** Certain attributes of the study such as the NNTC IDs of tissues, tissue request IDs, the tissues studied, study labels, and studied genes, proteins, and organisms are recorded here. By clicking on the arrow symbols the user can view the list of attributes
- **Contact(s):** The name of a contact person is given here, as well as an email address
- **Release Date:** The date that the study was released
- **Assay Downloads:** Here, the raw data files for each combination of measurement and technology as described earlier can be downloaded. By clicking on the ‘Raw Data’ link the user is taken to a new screen where the Excel files can be downloaded.

#### Gene search

Using the NCBI Gene Search tool, users can search the GEO profiles of only those experiments that use NNTC-derived tissues on the NCBI website (Figure 4c). The user can select up to 5 gene names from a drop-down list, which is dynamically populated with a list of genes from the HUGO database (28). A list of GSE experiment IDs are extracted and updated from the study descriptions in the MySQL database every day using a perl script via a cron job to facilitate this search.

## Conclusion and future directions

With the NNTC-DCC database, our hope is to assist neuroAIDS scientists in their research by publishing HIV-related studies along with their experimental datasets. Through this approach, researchers can build upon the results of others and reduce redundancy. We are currently working on integrating clinical data provided by the NNTC to the EMMES Corporation database into our database via a patient query form. This allows for the downloading of experimental and clinical datasets based on patient IDs. We are also planning on integrating further clinical datasets as provided by the NNTC sites. We will incrementally update the experimental data from additional studies as they are obtained.

As researchers continue to perform experiments on NNTC specimens (in addition to using the NNTC-DCC database), datasets will be continuously enriched. To create the database in its current form, the NNTC-DCC data curator personally requested each NNTC user to transfer through secure email their generated data in Excel format. In some cases (especially for studies with relatively large amounts of data), a secure FTP connection was used to share the data. In the future, the data curator will continue to play an important role, with regular correspondence with NNTC resource users. These steps have now been incorporated in the routine exchange process for those wishing to utilize NNTC specimens for their research. Users propose studies via the request form located at https://www.nntc.org/. Upon submission of the tissue or data request, the NNTC steering committee reviews it, and potentially suggests modifications, in order to ensure proper stewardship of the resource. Once a tissue request is approved, the samples are shipped from the appropriate NNTC site(s) to the researchers. As part of the signed specimen usage agreement, recipients agree to electronic transmission back to NNTC-DCC of all data generated from the use of NNTC specimens within one year of receipt. This transfer will be coordinated by the data curator, working with the investigator to receive the data and metadata and then integrating them into the database. As studies and analyses can take more than one year, the curator will work with the investigator to ensure transfer of all data, and appropriate embargoes on public data release until publication of the findings. This process will lead to growth of the database and accessibility of these important findings for the scientific community, enriching the field and enabling progress through data sharing.

## Supplementary Data

Supplementary data are available at *Database* Online.

Supplementary Data
