# Supplementary material for: The National NeuroAIDS Tissue Consortium (NNTC) Database: an integrated database for HIV-related studies
Source: Database (Oxford). 2015 Jul 30;2015:bav074. doi: 10.1093/database/bav074 (PMC4520230; doi:10.1093/database/bav074)
Supplement: Supplementary Data [file supp_2015_bav074_index.html]

The National NeuroAIDS Tissue Consortium (NNTC) Database: an integrated database for HIV-related studies — Supplementary Data 

# The National NeuroAIDS Tissue Consortium (NNTC) Database: an integrated database for HIV-related studies

## Supplementary Data

files

- Supplementary Data - xlsx file
